# Conservative management of a Cesarean scar ectopic pregnancy: a case report

**DOI:** 10.4076/1757-1626-2-7794

**Published:** 2009-08-18

**Authors:** Luce Tulpin, Olivier Morel, Cécile Malartic, Emmanuel Barranger

**Affiliations:** Obstetrics and Gynecology Unit, Lariboisière HospitalAPHP, 2, rue Ambroise Paré, 75010 ParisFrance

## Abstract

**Introduction:**

Cesarean scar pregnancy is the rarest kind of ectopic pregnancy. The immediate prognosis depends on the risks associated with uterine rupture and massive bleeding.

**Case presentation:**

A 32-year-old woman (gravida 2, para 1) presented with massive vaginal bleeding. A Cesarean scar pregnancy was diagnosed. She was treated by local methotrexate injection, followed by uterine artery embolization. Recurrence of bleeding necessitated two repeat embolizations. Hysteroscopy four months later revealed the presence of a uterine defect within the Cesarean section scar.

**Conclusion:**

Cesarean scar pregnancy should be diagnosed and treated as soon as possible to prevent severe complications and spare fertility.

## Introduction

Cesarean scar pregnancy is the rarest kind of ectopic pregnancy but, because of the increasing number of Cesarean deliveries over the last 30 years, its incidence has been rising (currently about 1/2000 normal pregnancies) [1]. In Cesarean scar pregnancy, the gestational sac is implanted in the myometrium at the site of a previous Cesarean section. Complications, such as uterine rupture and massive hemorrhage, may be life-threatening and impact negatively on future fertility. It is important to be able to diagnose the condition as early as possible in order to administer conservative treatment. We describe a case of Cesarean scar pregnancy treated by methotrexate (MTX) and multiple selective uterine artery embolizations (UAE).

## Case presentation

**Figure 1.**
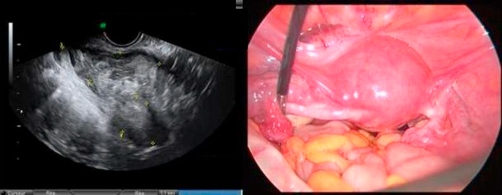
Diagnosis: **(Left)** 5-cm diameter cervico-isthmic gestational sac as seen on endovaginal ultrasonography; **(Right)** laparoscopic exploration.

A French Caucasian 32-year-old woman, gravida 2, para 1, with a history of Cesarean section, was admitted to hospital with uncontrollable vaginal bleeding and hypovolemic shock. Endovaginal ultrasonography revealed an ectopic pregnancy with a 5 cm-diameter cervico-isthmic gestational sac (Figure 1A). The serum beta-human chorionic gonadotrophin (β-hCG) level was 73,170 IU/mL. Because of the severity of bleeding and fear of uterine rupture, the patient underwent laparoscopic surgery. The diagnosis of Cesarean scar pregnancy was confirmed on pelvic exploration (Figure 1B). During laparoscopy, the amniotic fluid was sucked out, MTX (60 mg) was injected into the gestational sac, and intrauterine compression was achieved by introducing a urinary probe via the cervical canal. Bilateral UAE was performed after surgery and successfully halted vaginal bleeding.

Magnetic Resonance Imaging (MRI) on day 3 after UAE revealed that the gestational sac (6 cm in diameter) was implanted at the site of a previous Cesarean section scar and was still vascularized peripherally. On the other hand, the placenta was located within the uterine cavity and vascularization was much reduced. The β-hCG level on day 5 after UAE was 44,737 IU/mL. Because of the persistence of blood vessels around the gestational sac, a second, selective UAE procedure was performed 9 days after the first UAE. It resulted in considerable, but not complete, regression of vascularization as evidenced by MRI. The β-hCG level fell to 12,174 IU/mL. Vaginal bleeding ceased and the patient was discharged home. During follow-up, she remained clinically stable but serial ultrasound assessments showed a persistent 6-cm vascularized mass. The β-hCG level continued to fall.

However, 24 days after the first UAE, the patient was re-admitted to hospital with major vaginal bleeding requiring a blood transfusion. Since she remained hemodynamically stable, a third selective UAE was performed and accompanied, as before, by intrauterine compression. The patient complained of no pain and of only minimal bleeding after the procedure. Endovaginal color Doppler ultrasonography confirmed devascularization of the gestational sac.

**Figure 2.**
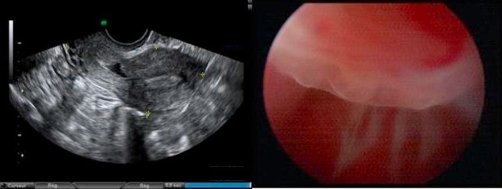
Uterine defect within the Caesarean section scar 4 months after treatment: **(Left)** endovaginal ultrasonography; **(Right)** hysteroscopy.

Four months after treatment, clinical and ultrasound results were stable (Figure 2A). Hysteroscopy to evaluate wound healing revealed the presence of a uterine defect within the Cesarean section scar (Figure 2B).

## Discussion

Cesarean scar pregnancy should be diagnosed as early as possible in order to avoid severe complications and provide conservative treatment. Most of the cases that have been reported were diagnosed early in the first trimester. The most common symptom is painless vaginal bleeding that may be massive. Diagnosis is difficult but often possible using endovaginal ultrasonography and color flow Doppler [1]. Proposed ultrasound diagnostic criteria, allowing a differential diagnosis with cervical ectopic pregnancy, are: (i) a gestational sac located between the bladder wall and the anterior isthmic portion of the uterus; (ii) no trophoblastic tissue visible in the uterine cavity and cervical canal; and (iii) clearly visible circular blood flow surrounding the sac [2-4]. When the pregnancy is not localized by ultrasonography, either laparoscopy or laparotomy can be used for the diagnosis.

Because of the risk of uterine rupture and uncontrollable bleeding, hysterectomy is indicated. However, several types of conservative treatment have been used: (i) dilatation, curettage and excision of trophoblastic tissues using laparotomy or laparoscopy [5,6]; (ii) local and/or systemic MTX administration [7]; (iii) bilateral hypogastric artery ligation, associated with dilatation and evacuation under laparoscopic guidance [8]; and (iv) selective UAE in combination with curettage and/or MTX injections [2,9]. UAE appears to prevent excessive bleeding and spare fertility.

Our patient received a local injection of MTX and underwent UAE. However, because vaginal bleeding recurred a couple of weeks after UAE, a second, selective UAE procedure was undertaken. This was possible as the patient was hemodynamically stable. Treatment depends on several criteria including hemodynamic status, severity of vaginal bleeding, presence of fetal cardiac activity, gestational age, and β-hCG level.

The immediate complications of Cesarean scar pregnancy are uterine rupture, severe bleeding, need for hysterectomy, and maternal morbidity. Long-term outcomes to be considered after conservative treatment are future fertility and recurrence of Cesarean scar pregnancy. A recent study of 29 successfully treated women with follow-up data reported favorable reproductive outcomes and an apparently low recurrence rate [10]. Out of the 24 women attempting to become pregnant, 21 conceived spontaneously (20 intrauterine pregnancies and one recurrent scar pregnancy). Thirteen of the 20 intrauterine pregnancies appeared normal; 9 were delivered by Cesarean section. The other 7 pregnancies ended in spontaneous abortions. Recently, a patient who conceived one year after medical and surgical treatment underwent a prophylactic Cesarean section in a medical center with UAE facilities because of a suspected risk of abnormal placental insertion [11].

Our patient underwent hysteroscopy 4 months after conservative treatment. The examination revealed the presence of a large uterine defect within the Cesarean section scar.

## Conclusion

To avoid severe maternal morbidity and spare fertility in patients with Cesarean scar pregnancy, minimally invasive surgery and/or MTX therapy, followed by selective, possibly repeat UAE, is indicated either as therapy to treat a symptomatic ectopic pregnancy (as in our patient) or as prophylaxis to prevent uncontrollable bleeding. A hysteroscopy should be performed a few months after treatment to evaluate the Cesarean section scar.

## Abbreviations

β-hCG: beta chorionic gonadotrope hormone
MRI: magnetic resonance imaging
MTX: methotrexate
UAE: selective uterine artery embolizations
